# Stiffener Design to Maintain Line Heating Efficiency during the Lifting Process Considering Phase Transformation

**DOI:** 10.3390/ma15010119

**Published:** 2021-12-24

**Authors:** Hong-Jun Noh, Hun-Bong Lim, Hee-Chan Yoon, Young-Hwan Han, Hyun-Ik Yang

**Affiliations:** 1 Department of Mechanical Engineering, Hanyang University, 55, Hanyangdaehak-ro, Sangnok-gu, Ansan 15588, Korea; nohoju86@hanyang.ac.kr (H.-J.N.); nike029@hanyang.ac.kr (H.-C.Y.); hyh1994@hanyang.ac.kr (Y.-H.H.); 2 Department of Mechanical Design Engineering, Myongji College, 134, Gajwa-ro, Seodaemun-gu, Seoul 03656, Korea; lhb1019@mjc.ac.kr

**Keywords:** equivalent thermal strain method, line heating, lifting, inherent strain

## Abstract

In the shipbuilding industry, welding is the main technique used to join steel structures. There is a lifting process, post-welding, that can eliminate the correction effect of line heating. Line heating is reperformed after the lifting process. This can significantly delay the ship assembly process. Herein, we present a design method for installing a permanent stiffener to avoid the disappearance of the line heating effect during the lifting process. The change in physical properties due to heating and cooling of the line heating is calculated. The limiting stress, at which the effect of the line heating completely disappears, based on the inherent strain theory, is obtained. The phase fraction by the cooling rate is calculated using the continuous cooling transformation diagram and the Kiustinen–Marburgerm equation. Physical properties affected by the phase transformation are calculated, considering the physical properties and fraction of each phase. The square plate theory and superposition principle are used to construct a local model, with a stiffener, of the ship block. The stress caused by the shape of the stiffener and the distance between the stiffeners were calculated for the local model. The calculated stress and the limiting stress were compared to determine, for the expected line heating efficiency, the most acceptable stiffener design. Finally, to confirm the elimination of the problem, the designed stiffener is analyzed using the finite element method.

## 1. Introduction

Welding causes deformation that is classified into in-plane or out-of-plane depending on its direction. Deformation reduces the strength of the structure and affects the assembly process, requiring a post-treatment process to remove it. Line heating is used as a post-treatment process to remove the deformation generated by welding. It offsets the previously produced welding deformation by inherent strain caused by heating the plate above a critical temperature. It is also used to create plates with a curvature. Line heating causes various effects such as temperature changes of plates due to heating and cooling and changes in physical characteristics by phase transformation.

Fujishiro et al. [1], who experimentally analyzed the sensitivities of the burner height, tip height, test piece thickness, oxygen pressure, and cooling methods, first studied line heating. Plate thickness had the greatest sensitivity amongst all the considered variables. Lim et al. [2] experimentally analyzed the changes in the physical properties caused by the heating and cooling process of line heating. They used a microscope and a tensile tester to confirm that the grains in the heat-affected zone (HAZ) were refined with ferrite and pearlite and that the yield and tensile strength were changed. Nomoto et al. [3] analyzed the deformation caused by line heating by dividing the cooling region into the imaging jet region, pool boiling region, and laminar flow region, and they considered changes in physical properties because of phase transformation. Hwang et al. [4] analyzed the generation of curved plates by line heating using the form of HAZ obtained experimentally, and they also considered changes in the physical properties caused by phase transformation. Biswas et al. [5] performed a transient thermal deformation analysis for line heating using a 3D finite element model considering the shape of the heat source and applied temperature-dependent convection and physical characteristics to the analysis model. Since transient thermal deformation analysis using the 3D finite element model requires a significant amount of time, a simple analysis method was developed to solve this problem. The inherent strain of the HAZ for the simple analysis method was defined by Ko [6], which was used to calculate an equivalent load for obtaining deformation by line heating. Ha [7,8] developed the strain direct boundary (SDB) method using the inherent strain and used it to predict the thermal deformation of unit specimens and large structures.

After the line heating, the ship block went through an assembly process using a crane. Choi et al. [9] analyzed the cable tension for each work step in the lifting process using four cranes and presented operation guidelines for workers. Muhammad et al. [10] constructed lug arrangement cases and compared the deformation of the ship block for each case to determine the best case. Based on the absolute nodal coordinate formulation, Ham et al. [11] developed a program to analyze the stress of a ship block generated during the lifting process by connecting the shell and rigid element with a ball or fixed joint. Lee et al. [12] suggested an optimal lug position for the stable lifting process of ship blocks using a multi-objective genetic algorithm.

Ha et al. [13,14] analyzed the stresses of a ship block during the lifting process using an elastic analysis considering changes in physical properties caused by line heating. They explained that the inherent strain due to the line heating is reduced by the tensile stress, which may cause the correction effect by line heating to disappear. They calculated the stress, based on the inherent strain theory, at which the correction effect completely disappears. They determined the ratio of the correction effect remaining after the lifting process, by comparing it with the stress calculated in the elastic analysis. They determined where to install the stiffener, but the stiffener type was not analyzed.

Both the location and the shape of the stiffener affect the stiffness of the ship block, causing differences in the ratio of the correction effect remaining after the lifting process. Therefore, the most acceptable shape and position of the stiffener must be determined. In addition, permanently installed stiffeners affect not only the design of ship blocks but also the ship outfitting design; hence, the specifications of the stiffener must be determined at the design stage itself. Therefore, in this study, we propose a method to determine the design parameters of a permanent stiffener, which would maintain the correction effect induced by line heating, during the lifting process. First, the magnitude of the tensile stress at which the correction effect induced by the line heating completely disappeared was calculated. To this end, the final phase fraction caused by heating and cooling was determined by considering the cooling rate and chemical composition of the carbon steel. In addition, the physical properties of the line heating position are determined by considering the yield strength of each phase and the volume expansion caused by the phase transformation to martensite. Next, a local model of the ship block was constructed based on plate theory to calculate the tensile stress caused by the lifting process. The upper and lower sides of the square plate were fixed, and the stiffeners were connected to the left and right sides. It was assumed that the stiffener was a beam element and was supported only for out-of-plane bending. The tensile stress, when gravity was applied, was calculated based on the density and thickness of the stiffener and the distance between the stiffeners. The calculated stress was compared with the tensile stress in which the correction effect completely disappeared to obtain the line heating efficiency. Specifications of the most acceptable stiffener were determined using the line heating efficiency. Finally, the finite element model of the ship block was constructed to obtain the tensile stress caused by the specifications of the stiffener. The specifications were verified for the expected line heating efficiency.

## 2. Procedures to Determine Specifications of the Stiffener

**Figure 1:**
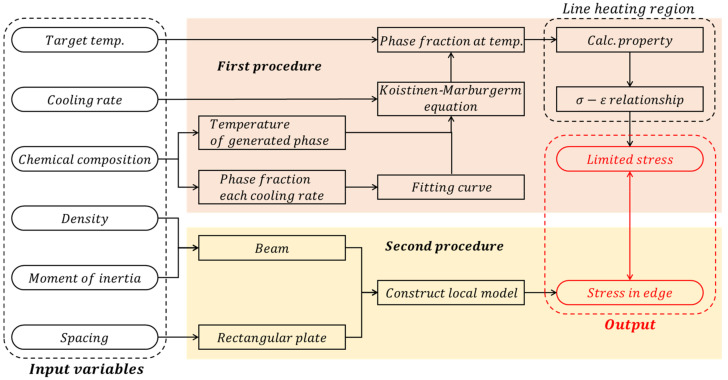
Procedures for determining the stiffeners.

The process for determining the specifications of the stiffener to maintain the correction effect of the line heating is divided into two procedures, as shown in Figure 1. The detailed procedure for each is described in Section 3 and Section 4, and a brief procedure is described in this chapter.

The first procedure is to obtain the stress where the correction effect by the line heating completely disappears based on the inherent strain theory. The input variables for this are the chemical composition of the material, the target temperature, and the cooling rate. Depending on the chemical composition, the temperature at which the generation of bainite, martensite, and pearlite starts and the temperature at which the generation of martensite and pearlite ends are calculated. The cooling rate at which the final phase fraction of bainite and martensite accounted for 100%, 50%, and 0% was calculated by the chemical component. In addition, the cooling rate at which the final phase fraction of martensite accounts for 100%, 90%, and 50% is calculated by the chemical component. Using the relationship between the cooling rate and the final phase fraction, the function to obtain the final phase fraction for arbitrary cooling rates is determined by curve fitting. The amount of the generated phase gradually decreases from the temperature at which a phase starts to be generated to the temperature at which it ends, and a phase fraction for an arbitrary temperature is calculated using the Koistinen-Marburgerm equation that can take this effect into account. To calculate the yield strength after line heating, the yield strength of each phase was calculated using the chemical composition of the material, and these were summed according to the calculated phase fraction. Martensite expands in volume into the body-centered tetragonal (BCT) structure during transformation and the linear strain rate is obtained by considering this expansion. The calculated linear strain was then differentiated to obtain the thermal expansion coefficient. The stress-strain relationship in the line heating area is determined using a disk-spring model based on the inherent strain theory. This process uses the previously calculated yield strength and the thermal expansion coefficient. Next, using the method proposed by Ha [14], the magnitude of the tensile stress at which the correction effect caused by the line heating completely disappears was obtained. In the second procedure, the tensile stress due to the lifting process is calculated by constructing a local model of the ship block based on the plate theory; the input variables in this calculation are the density of the stiffener, stiffener’s moment of inertia, and distance between stiffeners. The local model consists of a square plate and beams, and the size of the square plate is determined by considering the distance between the stiffeners. The beam was connected to the left and right sides of the local model, which was determined by the density and moment of inertia. Finally, the tensile stress of the edges induced by the lifting process was obtained in the local model. By comparing the tensile stress calculated in each process, the ratio at which the correction effect disappears by the lifting process is determined, and the optimal specifications of the stiffener are determined.

## 3. Inherent Strain of Line Heating Considering Phase Transformation

Line heating is a procedure to correct deformation by generating the inherent strain ($\epsilon^{*}$) because of heating and cooling. The inherent strain refers to the strain that is not recovered when structural restraints or loads are removed. During heating and cooling processes, in the region where line heating is applied, strains are generated, including elastic strain, plastic strain ($\epsilon^{p}$), thermal strain ($\epsilon^{th}$), and phase transformation strain ($\epsilon^{tr}$). 

**Figure 2:**
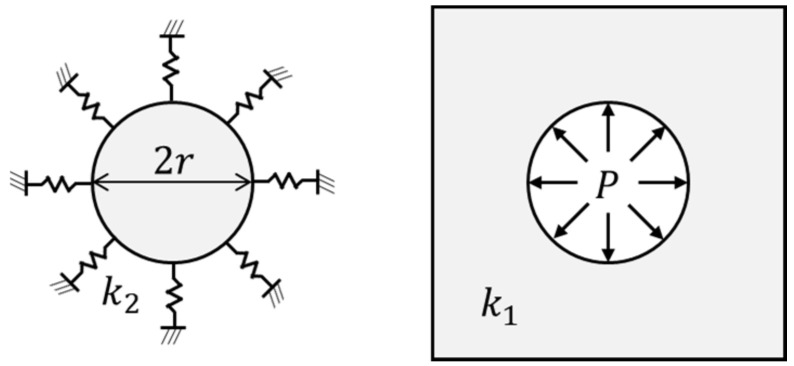
Disk-spring model.

**Figure 3:**
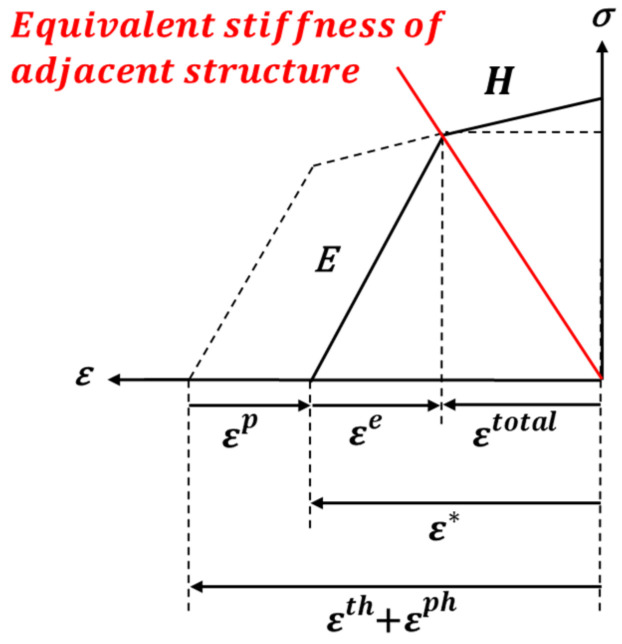
Relationship between stress and strain in the line heating area due to the equivalent stiffness of the adjacent structure.

The inherent strain excludes elastic strain ($\epsilon^{e}$) from total strain ($\epsilon^{total}$), as shown in Equation (1). The inherent strain due to line heating is obtained from the disk-spring model proposed by Ko [6]. The disk spring model has a plastic-elastic region where heating and cooling are applied and an elastic region around the line heating area, as shown in Figure 2. Equation (2) shows the result of the inherent strain calculated from Ko’s [6] model. E, k and ν are Young’s modulus, stiffness, and Poisson’s ratio of the line heating area ($E_{1},\;k_{1},\;\nu_{1}$) and its surrounding area ($E_{2},\;k_{2},\;\nu_{2}$), respectively, and $\sigma_{Y}$ is the yield strength of the line heating area. Figure 3 presents the stress–strain relationship of the line heating area constructed by the inherent strain in the reference model. According to the value of the inherent strain, the reference axis moves from the position before the line heating, and the stress and strain of the line heating area are determined based on the equivalent stiffness of the structure.

(1) $$\epsilon^{*}=\epsilon^{th}+\epsilon^{p}+\epsilon^{tr}=\epsilon^{total}-\epsilon^{e}$$

(2) $$\begin{array}{l} \varepsilon^{*}=-\frac{\sigma_{Y}}{E_{1}}\left(\frac{k_{1}}{k_{2}}+1\right)=-\frac{\sigma_{Y}}{E_{1}}\left(\frac{E_{1}}{E_{2}}\frac{1+\nu }{1-\nu }+1\right)\;\\ where\;\;k_{1}=\frac{E_{1}}{r\left(1-\nu_{1}\right)},\;k_{2}=\frac{E_{2}}{r\left(1+\nu_{2}\right)} \end{array}$$

When the local region is heated from room temperature to a temperature above *Ac*_1_ by the line heating, austenite begins to form by crystalline transformations, in which austenite has a high carbon solubility. Dissolved carbon starts to be released during the cooling process, and the result of the phase transformation depends on the amount of carbon released according to the cooling rate [15]. Thus, they exhibit have different stress–strain relationships, because the physical properties of each phase are different. Consequently, a difference occurs in the stress–strain of the line heating area with phase transformation. Therefore, the phase fractions from the heating and cooling processes were obtained and applied to the analytical model. During the heating process, if the amount of carbon is determined, the phase transform remains unchanged, and the temperature (*Ac*_1_) at which the transformation from ferrite to austenite begins the reference temperature for determining the inherent strain region [16]. During the cooling process, the amount of carbon released from austenite depends on the cooling rate, which is converted into martensite, bainite, and perlite. The reference temperature for determining the phase fraction according to the cooling rate during the cooling process can be calculated using Equations (4)–(8), where *Bs*, *Ms*, and *Fs* are the temperatures at which the bainite, martensite, and ferrite phases start to form, respectively; *Mf* is the temperature at which the formation of the martensite phase is terminated; *F*50 is the temperature at which 50% of the ferrite phase is formed [16,17].

(3) $$Ac_{1}=723-10.7Mn-16.9Ni+29.1Si+16.9Cr+290As+6.38W$$

(4) Bs=830−270C−90Mn−37Ni−70Cr−83Mo

(5) Ms=512−453C−16.9Ni+15Cr−9.5Mo+217C2−71.5CMn−67.6CCr−11.1Si

(6) Mf=Ms−215

(7) $$Fs=\left\{12log\left(Cn\right)+862\right\}+C\left\{50.5log\left(Cn\right)-341.8\right\}+Mn\left\{10.9log\left(Cn\right)-86.43\right\}$$

(8) $$F50=\left\{12log\left(Cn\right)+862\right\}+C\left\{50.5log\left(Cn\right)-341.8\right\}+Mn\left\{10.9log\left(Cn\right)-86.43\right\}$$

**Figure 4:**
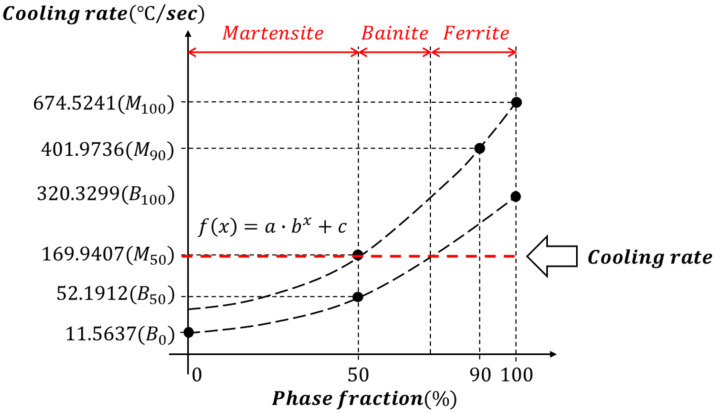
Phase fraction by the cooling rate.

The phase fraction according to the cooling rate in the continuous cooling transformation diagrams for engineering steel can be expressed through Equations (9)–(14), and these data are used as a reference to obtain the final phase fraction relationship curve for all cooling rates in the fitting [18]. As shown in Figure 4, the region on the right side of the line drawn by $B_{100}$, $B_{50}$ and $B_{0}$ is the phase fraction occupied by martensite and bainite at an arbitrary cooling rate, and the region on the left is the phase fraction occupied by ferrite. In addition, the area on the right side of the line drawn by $M_{100}$, $M_{90}$ and $M_{50}$ is the phase fraction occupied by martensite at an arbitrary cooling rate.

(9) $$B_{100}=\frac{1}{3600}10^{7.93-3.8C-1.07Mn-0.57Cr-1.58Mo-0.7Ni}$$

(10) $$B_{50}=\frac{1}{3600}10^{6.5-2.23\;C-0.86Mn-0.59Cr-1.6Mo-0.56Ni}$$

(11) $$B_{0}=\frac{1}{3600}10^{5.03-0.43\;C-0.49Mn-0.26Cr-0.38Mo-2\sqrt{Mo}-0.78Ni}$$

(12) $$M_{100}=\frac{1}{3600}10^{8.524-4.62\;C-1.05Mn-0.5Cr-0.66Mo-0.54Ni}$$

(13) $$M_{90}=\frac{1}{3600}10^{8.06-4.04\;C-0.96Mn-0.58Cr-0.97Mo-0.49Ni}$$

(14) $$M_{50}=\frac{1}{3600}10^{7.66-4.13\;C-0.86Mn-0.41Cr-0.94Mo-0.57Ni}$$

**Figure 5:**
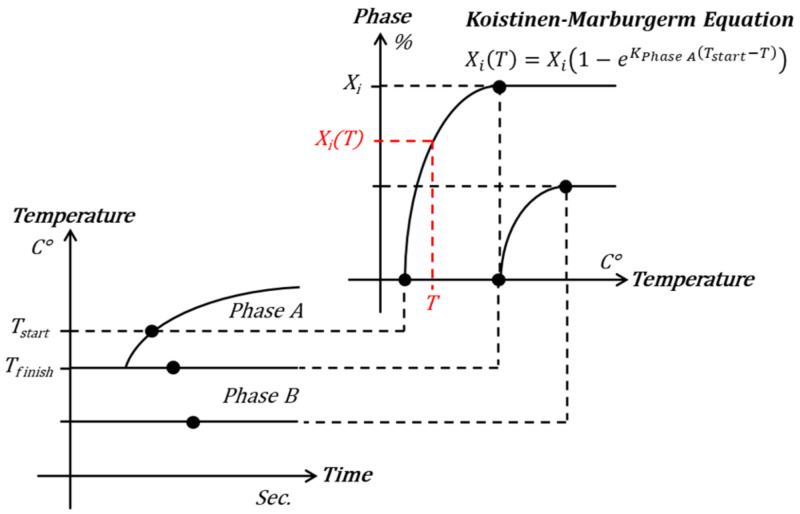
Phase fraction at any temperature, using the Kiustinen–Marburgerm equation.

Phase transformation proceeds rapidly at the temperature at which the phase transformation begins and then gradually slows down to reach the final phase fraction ($X_{i}$). Phase fraction ($X_{i}\left(T\right)$) at arbitrary temperature (T) can be expressed using the Kiustinen–Marburgerm equation [19] used to determine the ratio of the phases generated between the start and end temperatures ($T_{start},\;T_{finish}$). In this process, the transformation rate parameter ($K_{Phase\;A}$) is considered. Accordingly, the ratio of ferrite, bainite, martensite, and austenite to an arbitrary temperature is obtained as shown in Figure 5.

(15) $$f_{Cooling}\left(T\right)=\sum X_{i}\left(T\right)\cdot f_{i}\left(T\right)$$

**Figure 6:**
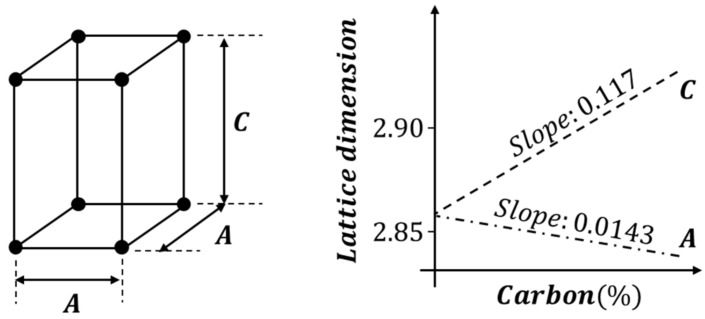
Lattice dimension by carbon ratio during martensite phase transformation.

Changes in physical properties due to phase transformation in the heating process depend only on changes in the temperature of the two phases, and general information regarding physical properties from *Ac*_1_ to *Ac*_3_ is a result of the mixed physical properties based on each fraction. Therefore, only the change in the physical properties due to the cooling process should be determined. Various phases are mixed, according to the cooling rate, and the physical properties ($f_{Cooling}$) of the mixing result can be calculated using Equation (15), considering the properties ($f_{i}$) and fractions ($X_{i}$) of each phase. Among the phases generated by cooling, martensite has a relatively significant impact on the final properties of the mixed-phase. The volumetric expansion during martensite’s phase transformation is due to the volume expansion (∆β) into the BCT structure, which is determined by the carbon content (C) as shown in Figure 6. The thermal strain ($\varepsilon_{cooling}$) according to the cooling temperature can be obtained, and the thermal expansion coefficient ($\alpha_{modified}$) can be obtained using differentiation as in Equations (16) and (17). The martensite yield stress is determined by the particle size of austenite, which is associated with the deoxidation of aluminum and the maximum temperature of austenite. The austenitic *ASTM* (American Society of Mechanical Engineers) grain size ($D_{g}$) number can be either coarse or fine, and the diameter of the grain can be obtained using Equation (18). The yield stress ($\sigma_{Y\_Martensite}$) of martensite was determined by substituting the calculated diameter of the grain. Consequently, the stress–strain relationship in the line heating area changes due to the difference in the yield strength and the thermal expansion coefficient induced by the phase transformation.

(16) $$\alpha_{modified}=\frac{d\varepsilon_{cooling}\left(T\right)}{dT}=\frac{d}{dT}\left(\int_{TM}^{T_{i}}\underset{i}{\sum }X_{i}\cdot \alpha_{i}\;dT+\varepsilon_{F\to M}^{ph}\cdot x_{M}\right)$$

(17) $$\varepsilon_{F\to M}^{ph}=\frac{∆\beta }{3}=\frac{1}{3}\left(\frac{\left(2.86+0.117C\right){\left(2.86-0.0143C\right)}^{2}}{2.86^{3}}-1\right)$$

(18) $$\sigma_{Y\_Martensite}=413+149.8\sqrt{C}+116.9\frac{\sqrt{C}}{\sqrt{D_{grain}\left(T_{max}\right)}}$$

**Figure 7:**
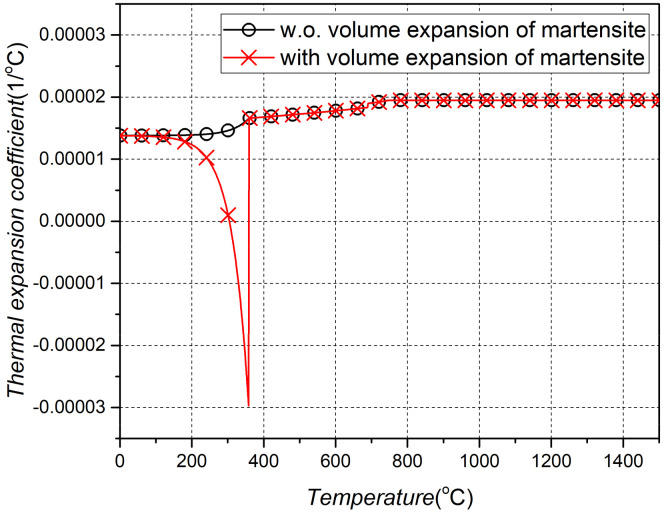
Thermal expansion coefficient after line heating.

Table 1 and Table 2 list the properties and chemical composition of carbon steel, respectively, which is the material used in this study. Figure 7 demonstrates the change in the thermal expansion coefficient when a material with a given composition undergoes a cooling process to generate martensite.

## 4. Determination of Stiffener to Maintain Weaving Effect under Lifting Process

Additional processes, such as lifting, may eliminate line heating effects that change the out-of-plane deformation of ship blocks. This is because tensile stress generated in the line heating area removes the inherent strain. Therefore, the stiffener must be installed permanently or temporarily to maintain the line heating effect during the lifting process.

**Figure 8:**
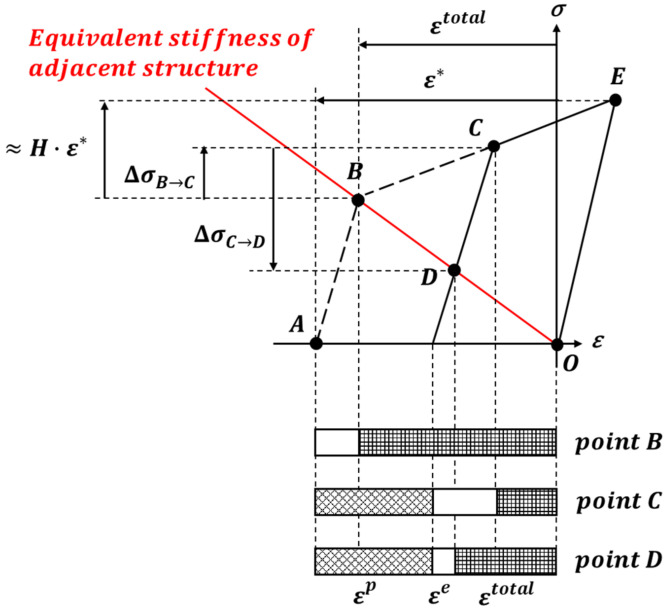
Changes in the stress and strain in the line heating area during lifting.

Based on the theory of inherent strain, Ha [13,14] used the stress–strain relationship and explained that the line heating effect disappears due to tensile stress, as shown in Figure 8. Therefore, we were able to obtain the stress at which the line heating effect completely disappeared. Changes in the stress and strain due to line heating and lifting are explained as shown in Figure 8. The thermal strain occurs in the line heating area because of heating and cooling processes (*Point A*). The total strain of the line heating area is determined by the equivalent rigidity of the structure (*Point B*). The stress and plastic strain of the line heating area is increased by lifting (*Point C*). Nevertheless, the total strain is reduced by the plastic strain and the total strain is not completely restored even after lifting is completed (*Point D*). If the stress increases to $H\cdot \varepsilon^{*}$ by lifting, the total strain completely disappears, thus losing the line heating effect (*Point O*).

**Figure 9:**
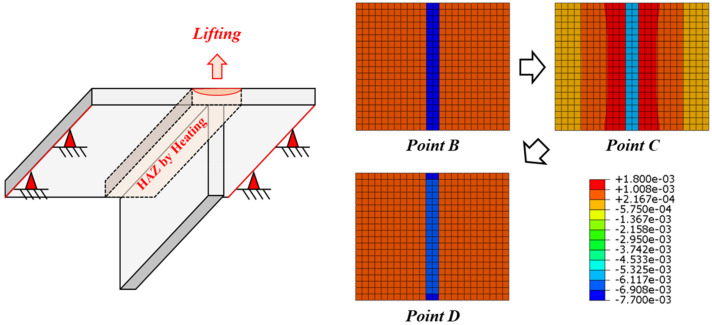
Inherent strain change in the line heating area by tensile stress.

This effect was confirmed using a model in which the flat plate and girder were connected (Figure 9). Both corners of the flat plate were fixed, and a load by lifting acted on the line heating area. The simulation showed that the inherent strain of the line hating area was reduced due to the lifting load (*Point B*→*Point C*). The reduced inherent strain did not recover even after the lifting load is removed (*Point C*→*Point D*). In calculating the line heating efficiency, the phase transformation due to heating and cooling was considered.

(19) $$\eta_{Heating}=\frac{\varepsilon_{C}^{Total}}{\varepsilon_{B}^{Total}}\propto \frac{H\cdot \varepsilon_{}^{*}-∆\sigma_{B\to C}^{}}{H\cdot \varepsilon_{}^{*}}=1-\frac{∆\sigma_{B\to C}^{}}{H\cdot \varepsilon_{}^{*}}$$

**Figure 10:**
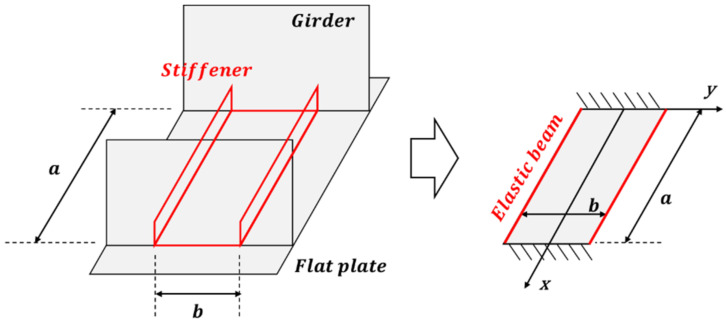
Simple model considering arrangement of the girder and plate.

The low line heating efficiency means that the inherent strain generated by the line heating is greatly reduced by the tensile stress, which means that the correction effect by the line heating is also greatly reduced. The minimum stress for the correction effect to completely disappear is $H\cdot \varepsilon^{*}$, and considering the properties of carbon steel, this value is 5.3070 MPa. As mentioned previously, a stiffener must be installed to maintain the line heating effect. The mathematical model that determines the shape of the stiffener and the distance between the stiffeners is defined as shown in Figure 10 and an equation is constructed to obtain the solution using the superposition method in the theoretical solution for each boundary condition (20). In Equation (20), D is the stiffness of the plate and q is a distributed load due to gravity. The stiffness of the plate is calculated using the plate thickness (h), Young’s modulus (E), and Poisson’s ratio (ν). The top and bottom corners of this model are constrained by fixed joints, and the left and right corners are connected by beams replacing the stiffener which has a moment of inertia (I). The connected beam only supports bending in the x-direction and does not support bending and twisting in other directions.

**Figure 11:**
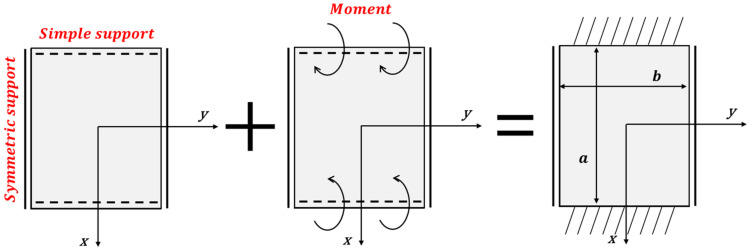
Implementation of a fixed support using the superposition method.

As shown in Figure 11, the results in which the top and bottom edges are fixed supported are obtained by overlapping the simple support result ($\omega_{1}$) with the result ($\omega_{2}$) of the moment. First, when the upper and lower edges are simply supported, a partial solution and homogeneous solution ($\omega_{p}$,$\omega_{h}$) can be obtained as described in Equation (21). Equation (24) expresses the solution to a given constituent equation and boundary conditions when the upper and lower edges are simply supported [20,21].

(20) $$\frac{\partial^{4}\omega_{1}}{\partial x^{4}}+2\frac{\partial^{4}\omega_{1}}{\partial x^{2}\partial y^{2}}+\frac{\partial^{4}\omega_{1}}{\partial x^{4}}=\frac{q}{D}$$

(21) $$\omega_{1}=\omega_{p}+\omega_{h}=\frac{4qa^{4}}{\pi^{5}D}\overset{\infty }{\underset{m=1,3,5,\cdots }{\sum }}\frac{1}{m^{5}}cos\frac{m\pi x}{a}+\overset{\infty }{\underset{m=1,3,5,\cdots }{\sum }}Y_{m}cos\frac{m\pi x}{a}\;where\;Y_{m}=\frac{qa^{4}}{D}\left(A_{m}cosh\frac{m\pi y}{a}+B_{m}\frac{m\pi y}{a}cosh\frac{m\pi y}{a}\right),\;D=\frac{h^{3}E}{12\left(1-\nu^{2}\right)}$$

(22) $${\left.\frac{\partial \omega_{1}}{\partial y}\right|}_{y=\pm b/2}=0,\;{\left.\omega_{1}\right|}_{x=\pm a/2}={\left.\frac{\partial^{2}\omega_{1}}{\partial x^{2}}\right|}_{x=\pm a/2}=0$$

(23) $$D{\left[\frac{\partial^{3}\omega_{1}}{\partial y^{3}}+\left(2-\nu \right)\frac{\partial^{3}\omega_{1}}{\partial x^{2}\partial y}\right]}_{y=\pm b/2}={\left.EI\frac{\partial^{4}\omega_{1}}{\partial x^{4}}\right|}_{y=\pm b/2}$$

(24) $$\begin{array}{l} \omega_{1}=\frac{qa^{4}}{D}\overset{\infty }{\underset{m=1,3,5,\cdots }{\sum }}\left(\frac{4}{\pi^{5}m^{5}}+A_{m}\cosh\frac{m\pi y}{a}+B_{m}\frac{m\pi y}{a}\sinh\frac{m\pi y}{a}\right)cos\frac{m\pi x}{a}\\ where\\ A_{m}=\frac{4}{m^{5}\pi^{5}}\frac{\nu \left(1+\nu \right)\sinh\alpha_{m}-\nu \left(1-\nu \right)\alpha_{m}\cosh\alpha_{m}-m\pi \lambda \left(2\cosh\alpha_{m}+\alpha_{m}\sinh\alpha_{m}\;\right)}{\left(3+\nu \right)\left(1-\nu \right)\sinh\alpha_{m}\cosh\alpha_{m}-{\left(1-\nu \right)}^{2}\alpha_{m}+2m\pi \lambda {\left(\cosh\alpha_{m}\right)}^{2}}\\ B_{m}=\frac{4}{m^{5}\pi^{5}}\frac{\nu \left(1-\nu \right)\sinh\alpha_{m}+m\pi \lambda \cosh\alpha_{m}}{\left(3+\nu \right)\left(1-\nu \right)\sinh\alpha_{m}\cosh\alpha_{m1}-{\left(1-\nu \right)}^{2}\alpha_{m}+2m\pi \lambda {\left(\cosh\alpha_{m}\right)}^{2}} \end{array}$$

As shown in Figure 11, the result of fixed support can be identified by using the superposition of the result of an acting moment ($M_{x}$) on the edge and the result of being simply supported. Because the solution for the simple support was already obtained, only the results of the moments acting on the top and bottom edges had to be acquired. When the moment acts on the top and bottom edges, the boundary conditions are defined in Equation (25) and a solution satisfying the boundary condition is defined by Equation (26).

(25) $$-D{\left.\frac{\partial^{2}\omega_{2}}{\partial y^{2}}\right|}_{y=\pm b/2}=M_{x}=\sum E_{m}sin\frac{m\pi y}{b}$$

(26) $$\omega_{2}=\overset{\infty }{\underset{m=1,3,5,\cdots }{\sum }}\left(C_{m}\cosh\frac{m\pi x}{b}+D_{m}\frac{m\pi x}{b}\sinh\frac{m\pi x}{b}\right)sin\frac{m\pi y}{b}$$

(27) $${\left.\frac{\partial \left(\omega_{1}+\omega_{2}\right)}{\partial x}\right|}_{x=\pm a/2}=0$$

Because the upper and lower edges are fixed, the solution derived by superposition must satisfy Equation (27). Using Equation (27), the unknown coefficient of Equation (25) can be solved to calculate the moments of the upper and lower edges. Thus, the magnitude of tensile stress ($\sigma_{x}$) can be obtained using the calculated moments.

(28) $$\sigma_{x}=M_{x}\frac{6}{h^{2}}$$

A stiffener for maintaining line heating efficiency should be installed in a ship block composed of flat plates and girders. The line heating efficiency by the stiffener installed can be obtained using the tensile stress of Equation (28). When the distance between the girders is 3000 mm, the magnitude of tensile stress by the cross-sectional shape, material, distance, etc. of the stiffener was analyzed. Finally, the line heating efficiency for each variable was obtained using the tensile stress. This analysis was based on the above variables is undertaken by using the dimensionless stiffness ratio of the flat plate and stiffener.

**Figure 12:**
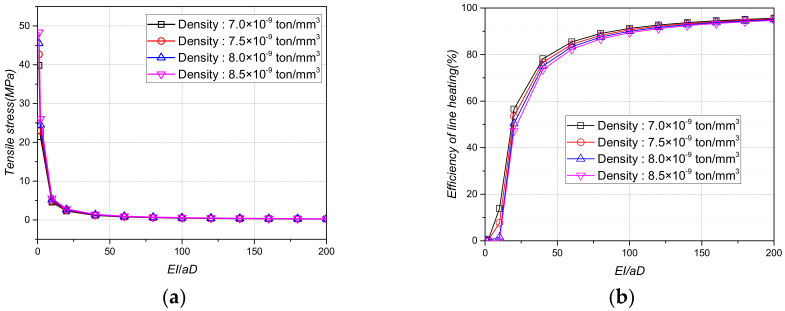
Tensile stress and line heating efficiency by stiffness ratio of plate and beam according to the density of the plate. (**a**)Tensile stress by density of the plate; (**b**) Line heating efficiency by density of the plate.

**Figure 13:**
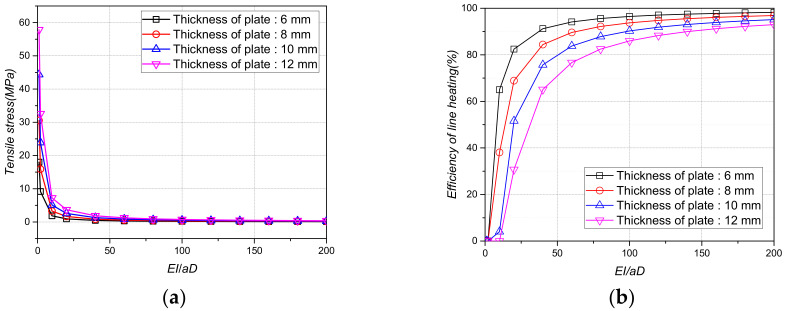
Tensile stress and line heating efficiency by stiffness ratio of plate and beam according to the thickness of the plate. (**a**) Tensile stress by thickness of the plate; (**b**) Line heating efficiency by thickness of the plate.

**Figure 14:**
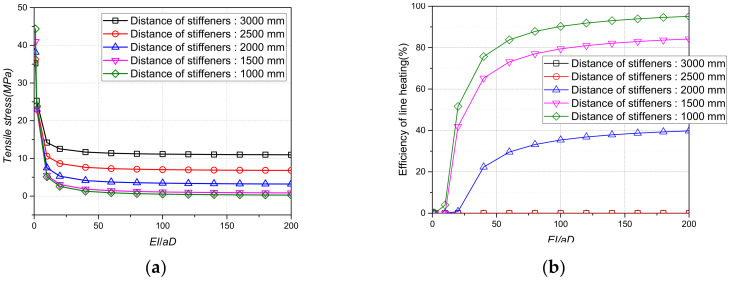
Tensile stress and line heating efficiency by stiffness ratio of plate and beam according to distance of stiffeners. (**a**)Tensile stress by thickness of the plate; (**b**) Line heating efficiency by distance of plates.

Figure 12, Figure 13 and Figure 14 show the tensile stress and line heating efficiency based on the density and thickness of the stiffener and the distance between the stiffeners using the stiffness ratio. As the stiffness ratio increased, the magnitude of the tensile stress decreased, and the line heating efficiency increased. In addition, as the stiffness ratio increased, the magnitude of the change in the tensile stress and the line heating efficiency gradually decreased. Even if the density of the stiffener increased from 7.0 × 10^−9^ tons/mm^3^ to 8.5 × 10^−9^ tons/mm^3^, the differences in the tensile stress and line heating efficiency were not significant. However, as the distance between the stiffeners increases from 1000 mm to 3000 mm, the tensile stress and heating efficiency change more significantly than the density. To maintain a line heating efficiency of at least 80% after lifting, the distance between the stiffeners must be greater than 1500 mm and the stiffness ratio between the stiffeners and the flat plate should be greater than 100.

## 5. Discussion

**Figure 15:**
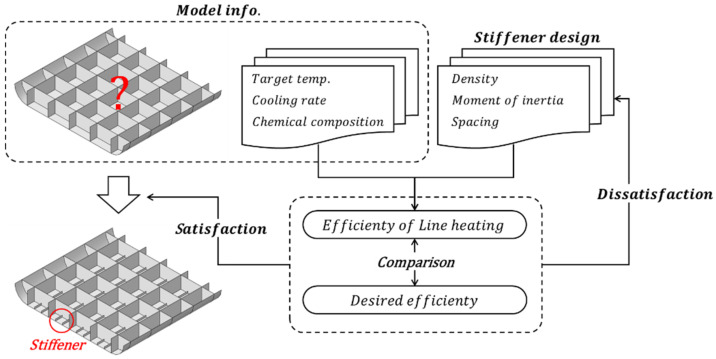
The procedure for determining the stiffener of the ship block.

Line heating is performed to correct out-of-plane deformation, and the resulting correction effect may disappear during the lifting process. To prevent this, a permanent stiffener is introduced, and the procedure for determining the permanent stiffener using the local model of the ship block was described in the previous chapters. As shown in Figure 15, the magnitude of the correction effect maintained by the stiffener is defined by the efficiency of the line heating, which can be compared with the desired efficiency to determine the specifications of the stiffener.

**Figure 16:**
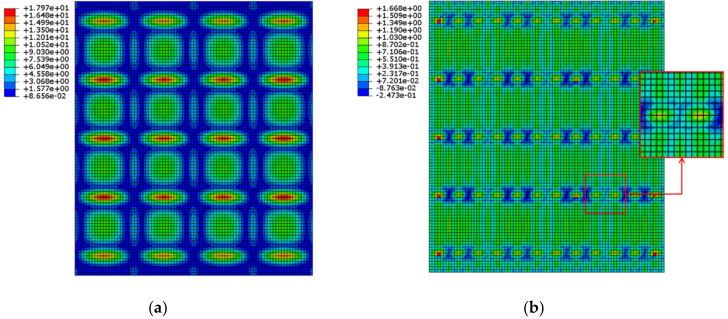
Tensile stress on the plate of the ship block by presence or absence of a stiffener. (**a**) Tensile stress without stiffener; (**b**) Tensile stress with stiffener.

The ship block model shown in Figure 15 was used to confirm the results of the local model. The length and width of the ship block model are 12 m and 15.6 m, respectively, and the height is 1.8 m. The thickness of the plate, made of carbon steel, excluding the stiffener was 10 mm. The finite element model was constructed using the S4R element to perform deformation analysis of the ship block caused by the lifting process. In the case of without stiffener, the tensile stress of the plate in the ship block is up to 17.97 MPa as shown in Figure 16a. It can be inferred that the calculated stress effect is greater than the tensile stress, and hence, the correction effect of line heating has completely disappeared owing to the lifting process. Figure 14b shows that the stiffness ratio between the plate and the stiffener should be 50 to maintain the line heating efficiency at 80%, and the thickness and height of the stiffener were determined to be 10 mm and 255 mm, respectively. A deformation analysis was performed on the ship block model with a stiffener, and it was confirmed that the maximum stress of the plate was 1.135 MPa, except at the location of stress concentration due to the connection of the lifting wire. The line heating efficiency calculated using the obtained stress was 78.61%, which was similar to the results of the local model.

The local model was used to determine the parameters of a permanent stiffener to maintain the correction effect made by the line heating of large models, such as ship blocks. Nowadays, ship blocks tend to be larger because of new construction methods and on-dock efficiency. The efficient design of larger ship blocks is possible using the local model proposed herein. As explained, the girder and frame connected to the plate were assumed to be rigid because of their relatively high rigidity against bending when the local model was constructed. However, as the torsion and bending of the girder and the frame cannot be ignored in the megaton-unit scale of a large model, additional research is needed to consider this situation. In addition, since the cooling rates of regions that undergo cooling and heating are not the same, various phases may occur in each region, resulting in differences in mechanical properties. Therefore, a more accurate solution can be obtained by analyzing the stress of phase generation for each cooling rate, in the line heating area, using the algorithm proposed by Bartel [22] and correcting the mechanical properties calculated by the inherent strain theory. Among the steel used as shipbuilding materials, there is a thermo-mechanical control process (TMCP) steel. This material forms bainite in the final phase by accelerating cooling after roll pressing through temperature control. Between the two solid phases constituting such steel, a nanometer-sized molten interface occurs at a temperature lower than a single melting temperature. This molten interface eliminates the consistency of the interface and relieves stress. Therefore, the magnitude of the maximum tensile stress, in which the correction effect completely disappears due to the lifting process, can be increased, and accurate results can be derived through Roy’s proposed method [23,24] regarding the energetics and kinematics of non-equilibrium interfacial nanoscale melt.

## 6. Conclusions

The line heating effect disappears in subsequent processes, such as lifting. In this study, we investigated why this occurs and how to maintain its correction effect. The phase fraction change is caused by the heating and cooling process of the line heating, and the phase fraction is calculated in consideration of the chemical composition and the cooling rate. Because the phases constituting the materials had different material properties, the material properties after the line heating process were calculated using the result of the final phase fraction. Accordingly, the difference in the phase fraction based on the cooling rate was investigated, and the Kiustinen–Marburgerm equation was used to derive the phase fraction for an arbitrary temperature during the cooling process. Consequently, the changed physical properties after line heating were obtained for the iron material with a given composition, and the stress–strain relationship in the line heating area was derived. It was confirmed that the correction effect induced by the line heating completely disappeared when tensile stress 5.3070 MPa was applied to the given carbon steel. Therefore, the correction effect remains only when the tensile stress generated by the lifting process is smaller than this value. Therefore, stiffeners were installed, and a simple mathematical model was devised to define the installation standards of these stiffener materials. The presented model had the shape of a square plate and beams that simulated stiffeners were connected. From this model, the tensile stress for the given gravity was obtained, and the effects of the thickness and density of the plate were analyzed, as well as the spacing between the stiffeners and the shape of the stiffener. The effects of the density and thickness of the plate on the tensile stress were low compared to the effects of the distance between the stiffeners. The line heating efficiency for each variable was confirmed through the stiffness ratio between the plate and the stiffener. In addition, the result of the local model confirmed that when the distance between the stiffeners is 1000 m, the stiffness ratio between the plate and the stiffener must exceed 50 to maintain the correction effect caused by the line heating to at least 80%. A finite element model for the ship block was constructed and compared to confirm the results of the local model. In addition, when a stiffener with a stiffness ratio of 50 was installed on the plate, the maximum tensile stress reached 1.135 MPa. The line heating efficiency at this is 78.61%, which is similar to the line heating efficiency calculated in the local model.

## Figures and Tables

**Table 1:** Composition of carbon steel.

Element.	Carbon (*C*)	Silicon (*Si*)	Tungsten (*W*)	Chromium (*Cr*)	Nickel (*Ni*)	Manganese (*Mn*)	Niobium (*Nb*)	Titanium (*Ti*)	Molybdenum (*Mo*)
Content	0.35	0.87	0.01	0.10	0.03	0.43	0.01	0.04	0.00

**Table 2:** Material Property of carbon steel.

Property	Carbon Steel
$\mathrm{Specific}\;\mathrm{gravity},\;g/{\mathrm{cm}}^{3}$	7.8
Melting point, ℃	1523
Tensile strength, MPa	535
Yield strength, MPa	414

## Data Availability

The data presented in this study are available on request from the corresponding author.

## References

[1] Hashimoto T., Fujishiro Y. (1958). An Experient of line heating designed with the table orthogonal array L32. J. Zosen Kiokai.

[2] Lim H.K., Lee J.S. (2017). On the material properties of shell plate formed by line heating. Int. J. Nav. Archit. Ocean Eng..

[3] Nomoto T., Jang C.D., Ha Y.S., Lee H.W., Ko D.E. (2011). A study on the simulation of water cooling process for the prediction of plate deformation due to line heating. Int. J. Ocean Syst. Eng..

[4] Hwang S.Y., Park K.G., Heo J.Y., Lee J.H. (2021). Thermal strain-based simplified prediction of thermal deformation caused by flame bending. Appl. Sci..

[5] Biswas P., Mandal N.R., Sha O.P. (2007). Three-dimensional finite element prediction of transient thermal history and residual deformation due to line heating. Proc. Inst. Mech. Eng. Part M J. Eng. Marit. Environ..

[6] Ko D.E. (1998). A Study on the Prediction of Deformations of Plates Due to Line Heating Using a Thermal Elasto-Plastic Analysis Model. Ph.D. Thesis.

[7] Ha Y.S. (2011). A study on weldment boundary condition for elasto-plastic thermal distortion analysis of large welded structures. J. Weld. Join..

[8] Ha Y.S. (2013). Analytical methodology obtaining an optimal welding sequence for least distortion of welded structure. J. Weld. Join..

[9] Choi K.S., Kim D.J. Lifting analysis for heavy ship hull blocks using 4 cranes. Proceedings of the Fourteenth International Offshore and Polar Engineering Conference.

[10] Muhammad N.M., Septia H.S., Dony S., Rizky C.A., Satriyo R. (2018). Structural analysis on the block lifting in shipbuilding construction process. MATEC Web Conf..

[11] Ham S.H., Roh M.I. (2021). Time-domain structural analysis during block turnover and lifting using 2D flexible multibody dynamics. Mar. Struct..

[12] Lee S.M., Roh M.I., Kim K.S., Ham S.H. (2018). Optimum design of lug arrangement based on static and dynamic analyses for block lifting. J. Ship Prod. Des..

[13] Ha Y.S., Won S.H., Yi M.S. (2008). A study for remained efficiency of correction heating after block lifting. Spec. Issue Soc. Nav. Archit. Korea.

[14] Ha Y.S. (2009). Method for Preventing Deformation Using Analysis Method Which Can Calculate Preservation Efficiency of Back Surface Heating Effect. Korea Patent.

[15] Thelning K.E. (2013). Steel and Its Heat Treatment.

[16] Andrews K.W. (1965). Empirical formulae for the calculation of some transformation temperatures. J. Iron Steel Inst..

[17] Payson P., Savage C.H. (1994). Martensite Reactions in Alloy Steels. Trans. Am. Soc. Met..

[18] Atkins M. (1977). Atlas of Continuous Cooling Transformation Diagrams for Engineering Steels.

[19] Deng D., Tong Y., Ma N., Murakawa H. (2013). Prediction of the residual welding stress in 2.25Cr-1Mo steel by taking into account the effect of the solid-state phase transformations. Acta Metall. Sin..

[20] Timoshenko S. (1959). Theory of Plates and Shells.

[21] Batista M. (2010). Uniformly Loaded Rectangular Thin Plates with Symmetrical Boundary Conditions. arXiv.

[22] Bartel T., Guschke I., Menzel A. (2019). Towards the simulation of Selective Laser Melting processes via phase transformation models. Comput. Math. Appl..

[23] Roy A.M. (2021). Formation and stability of nanosized, undercooled propagating intermediate melt during β→δ phase transformation in HMX nanocrystal. EPL.

[24] Roy A.M. (2021). Energetics and kinematics of undercooled nonequilibrium interfacial molten layer in cyclotetramethylene-tetranitramine crystal. Phys. B Condens. Matter.

